# Digitalizing a Brief Intervention to Reduce Intrusive Memories of Psychological Trauma: Qualitative Interview Study

**DOI:** 10.2196/23712

**Published:** 2021-02-22

**Authors:** Beau Gamble, Katherine Depa, Emily A Holmes, Marie Kanstrup

**Affiliations:** 1 Department of Psychology Uppsala University Uppsala Sweden; 2 Department of Clinical Neuroscience Karolinska Institutet Stockholm Sweden

**Keywords:** digital intervention, remote delivery, intrusive memories, psychological trauma, qualitative feedback, cognitive science, posttraumatic stress disorder

## Abstract

**Background:**

The COVID-19 pandemic has escalated the global need for remotely delivered and scalable interventions after psychological trauma. A brief intervention involving a computer game as an imagery-competing task has shown promising results for reducing the number of intrusive memories of trauma—one of the core clinical symptoms of posttraumatic stress disorder. To date, the intervention has only been delivered face-to-face. To be tested and implemented on a wider scale, digital adaptation for remote delivery is crucial. An important first step is to develop digitalized intervention materials in a systematic way based on feedback from clinicians, researchers, and students in preparation for pilot testing with target users.

**Objective:**

The first aim of this study is to obtain and analyze qualitative feedback on digital intervention materials, namely two animated videos and two quizzes that explain the target clinical symptoms and provide intervention instructions. The second aim is to refine the digitalized materials based on this feedback.

**Methods:**

We conducted semistructured interviews with 12 participants who had delivered or had knowledge of the intervention when delivered face-to-face. We obtained in-depth feedback on the perceived feasibility of using the digitalized materials and suggestions for improvements. Interviews were assessed using qualitative content analysis, and suggested improvements were evaluated for implementation using a systematic method of prioritization.

**Results:**

A total of three overarching themes were identified from the data. First, participants were highly positive about the potential benefits of using these digital materials for remote delivery, reporting that the videos effectively conveyed key concepts of the symptom and its treatment. Second, some modifications to the materials were suggested for improving clarity. On the basis of this feedback, we made nine specific changes. Finally, participants raised some key challenges for remote delivery, mainly in overcoming the lack of real-time communication during the intervention.

**Conclusions:**

Clinicians, researchers, and clinical psychology students were overall confident in the use of digitalized materials to remotely deliver a brief intervention to reduce intrusive memories of trauma. Guided by participant feedback, we identified and implemented changes to refine the intervention materials. This study lays the groundwork for the next step: pilot testing remote delivery of the full intervention to trauma survivors.

## Introduction

### Background

Most people will experience a psychologically traumatic event in their lifetime, that is, experience or witness actual or threatened serious injury, sexual violence, or death (eg, traffic accidents, assaults, war, or natural disasters) [1,2]. A significant proportion of those who experience traumatic events (about 1 in 4 [3,4] but more for some trauma types [5,6]) will develop posttraumatic stress disorder (PTSD). One of the main criteria of PTSD is the presence of intrusion symptoms such as intrusive memories of the traumatic event [2]. Although there are evidence-based treatments for PTSD, the clinical reality is that globally, most individuals do not receive psychological interventions of any kind after trauma [7]. Multiple barriers impede access to psychological treatment, such as geographical constraints, few or no treatment providers, and stigma surrounding help-seeking [7]. Thus, there is an urgent need for innovative, scalable interventions that can be readily administered to trauma survivors [8]. Advancing remote delivery of psychological interventions is also a significant step in the context of the ongoing COVID-19 pandemic, which has escalated the need for trauma treatments for vulnerable groups such as patients and health care staff [9]. It is essential to develop interventions that can be delivered safely for both target users and clinicians/researchers across the globe.

Recent clinical guidelines for PTSD highlight the utility of targeting single symptoms [3,4], and we have proposed targeting *intrusive memories of trauma*, one of the core clinical features of PTSD [2,10,11]. Intrusive memories are memories of traumatic event(s) that involuntarily and recurrently spring to mind, bringing back sights, sounds, or smells; evoking strong emotions; and hijacking attention [11]. Intrusive memories commonly occur as visual imagery [10], for example, after a car crash, a patient might report terrifying visual memories of blood and glass smashing. Intrusive memories of trauma can be highly distressing, are associated with functional impairment in daily life (eg, concentration difficulties) [12], and are central to the development of PTSD [13].

### A Novel Intervention to Reduce Intrusive Memories

Building on laboratory findings using analog trauma (ie, stressful film clips) [14,15], our group has developed a brief cognitive intervention involving a computer game as an imagery-competing task to reduce and prevent the number of intrusive memories of real-world trauma. The intervention is based on translational work combining the cognitive neuroscience of memory with intrusive memories of trauma [16,17] (for a detailed theoretical rationale, see Iyadurai et al [11]). The intervention first involves a brief reminder cue to activate the trauma memory in working memory and, for older memories, a 10-min time gap for the memory to become malleable [16]. Participants then play the computer game *Tetris*, a visuospatially demanding task, for at least 20 min using specific instructions for *mental rotation* to maximize the visual working memory load [17]. The intervention is thought to disrupt the (re)consolidation of traumatic memories, thereby reducing their tendency to intrude in everyday life [18].

Clinical studies have shown preliminary evidence that the intervention may lead to a reduced number of intrusive memories, reported in a daily diary over the course of 1 week, when compared with participants who did not use the intervention (treatment as usual or control task) [19-21] or compared with a baseline number of intrusive memories per week [22,23]. These studies included women who had just gone through a traumatic childbirth (emergency cesarean section) [19], patients recently admitted to the emergency department [20,21], inpatients with complex PTSD [22], and traumatized refugees [23]. So far, these studies have involved face-to-face delivery of the intervention by trained clinicians, researchers, and assistants. However, it is clear that there is great public interest in an *accessible* version of the intervention. After the recent explosion in Lebanon, more than 5000 people commented in a web-based forum about using *Tetris* and other games to alleviate distressing imagery [24]. For this intervention to be tested at scale and be truly accessible to many individuals over a wide geographic area, materials must now be adapted for remote digital delivery [25].

### This Study

Remote delivery of digital interventions comes with several challenges, such as generally high attrition [26] and low rates of participant engagement (including recent digital interventions for PTSD) [27], thereby requiring the need for careful and thoughtful intervention development. Frameworks such as the person-based approach (PBA) [28] emphasize the importance of incorporating qualitative user feedback in the iterative development of an intervention. To lay the groundwork for such pilot testing with target users (ie, trauma survivors), it is crucial to first obtain feedback on the digital intervention materials from clinicians, researchers, and students. This approach fits within the broader framework of patient and public involvement (PPI), which can include asking those who are in the same research team for constructive feedback on drafts of intervention materials [29]. The next step is to systematically evaluate the feedback and implement the top-priority changes to the materials. These initial refinements are especially important before piloting an intervention to groups such as trauma survivors, where poorly designed materials could be not only ineffective but inadvertently distressing (eg, if the materials trigger intrusive memories). Thus, the aims of this study are two-fold: (1) obtain and analyze qualitative feedback on digitalized intervention materials, namely, animated videos and quizzes that explain the target symptom and provide intervention instructions and (2) systematically refine the materials based on this feedback.

## Methods

### Design

We followed guiding principles for the development of the intervention materials, in line with PBA [28] (Multimedia Appendix 1). Qualitative feedback on the materials was obtained from nontarget users, within the framework of PPI [30], and proposed changes systematically evaluated for implementation using the Must have, Should have, Could have, Would like (MoSCoW) method of prioritization [31].

### Recruitment

We purposively recruited [32] a sample of researchers, clinicians, and clinical psychology students from an international research training group that regularly met to discuss the intervention and from the same research laboratory that the authors are a part of. Individuals were contacted by KD and invited to participate; all who were contacted accepted.

### Procedure

Semistructured interviews were carried out by the first author (KD), who had recently joined the research team and was not involved in the creation of the videos. We created an interview guide (Multimedia Appendix 2) that contained several open-ended questions (eg, *Please describe if you found anything to be helpful or unhelpful, and if so how?*) and a brief script to inform participants of the purpose of the interview (ie, to help design a digital, remote version of the intervention). Links to the videos and quizzes were sent to the participants before or during the interview. After watching the videos and completing the quizzes, the participants were asked to share their thoughts on the materials, suggestions for improvements, and any concerns they had in delivering a remote version of the intervention. Interviews were conducted over a private video call via Zoom (version 4.6.9) and lasted 8 to 32 minutes. Interviews were recorded, and verbal consent was obtained beforehand. Participants were not required to fill in an informed consent form; data collected from participants who were also colleagues involved in the same research team did not need to sign informed consent as part of PPI nor was ethical approval required [33].

### Materials

Videos can increase the accessibility and scalability of mental health treatments, which otherwise face barriers to access [34]. To move toward remote delivery of the intervention, we created two animated videos that explain the target symptom and provide instructions for the intervention. Video scripts were based on existing study protocols for in-person delivery of the intervention [25] and were designed and animated by a local artist [35] in collaboration with the core intervention development team (EH, MK, and BG). We followed recommendations on how to depict mental health images in a culturally sensitive way [36] and aimed for the videos to be applicable to a range of individuals with trauma history, for example, by featuring minimal text and a gender-neutral, nonstereotyped character. Materials were initially created in English, with plans to adapt them to other languages.

**Figure 1:**
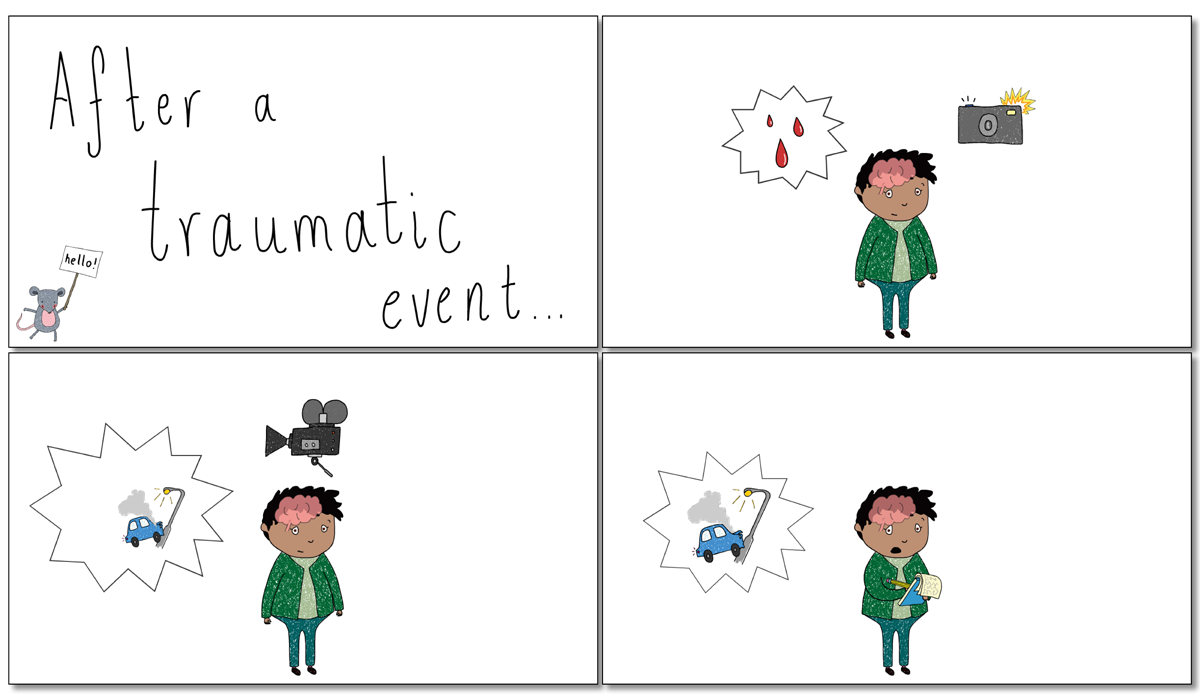
Screenshots from the “What are Intrusive Memories?” video, which explains the target symptom of the intervention in an accessible way.

**Figure 2:**
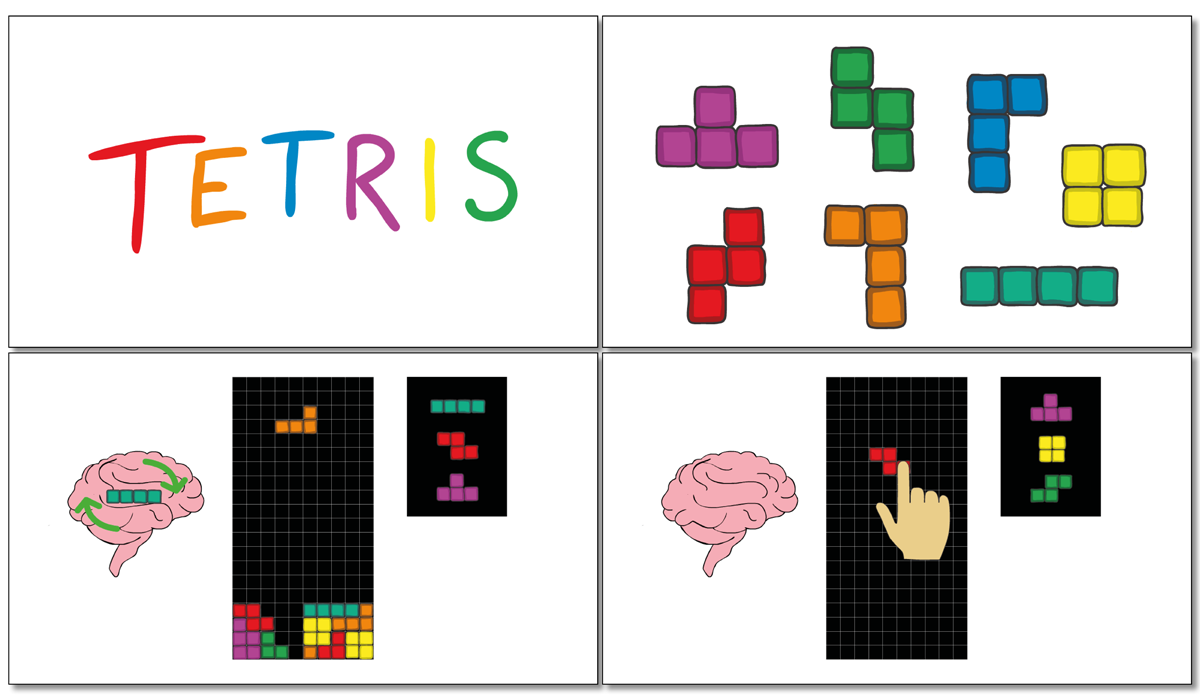
Screenshots from the “How to Play Tetris” video, which instructs participants on how to complete the imagery-competing task in the specific way required (eg, by emphasizing "mental rotation").

The first video (“What Are Intrusive Memories?”) defines and describes intrusive memories of trauma, the target symptom of the intervention (3 minutes 17 seconds; screenshots in Figure 1). The video explains, for example, that intrusive memories normally take the form of a visual image or movie clip in the mind’s eye and that they are *not* the same as rumination or deliberate recall (eg, *they are not the same as deliberately choosing to think about the event*). The second video (“How to Play Tetris”) describes how to complete the imagery-competing task in accordance with the intervention protocol (2 minutes 57 seconds; Figure 2). For example, the video explains how to use *mental rotation* to visualize and plan ahead, which is thought to be crucial for the intervention to work.

A total of two quizzes, each containing four questions relating to one of the videos, were created using Survey Monkey. The quizzes were designed to check the target user’s understanding of the video content (ie, what intrusive memories are and instructions for Tetris gameplay), which would typically be covered in a face-to-face session by the researcher asking the participant to summarize. The quizzes also aim to boost target user understanding and later recall of the content [37]. They were designed to be brief and with the goal that they could be completed in a few minutes. In a target user setting, the quizzes would be given immediately after users have been presented with the videos.

### Data Analysis

Interviews were transcribed verbatim and analyzed using qualitative content analysis [38]. Codes (Multimedia Appendix 3) and themes were initially extracted by KD using QDA Miner Lite (version 2.0.7) and iteratively renamed and restructured after input from the research team. Suggested changes to the materials were evaluated using a modified *MoSCoW* method such that changes were prioritized as Must have, Should have, Could have, or Would like [29]. For this step, the research team evaluated whether a suggested change would likely make a meaningful difference to intervention delivery, in line with our guiding principles, and if the change was feasible based on available resources [29]. Only changes prioritized as *Must have* (ie, both important and feasible) were implemented.

## Results

### Participants

All the participants (N=12) were female. The mean age of the sample was 31.3 years (SD 7.5). Participants described their ethnicity as Swedish (n=3), Swedish/European (n=1), Italian (n=1), Mixed/White and Asian (n=1), White/European (n=1), Icelandic/Caucasian (n=1), Irish/Caucasian/White (n=1), White/British (n=1), White/Caucasian Australian (n=1), and Caucasian (n=1). The sample comprised 6 researcher clinicians, 3 clinical psychology students, 1 researcher, 1 clinician, and 1 intern. Across the sample, participants worked with a broad range of groups with a history of trauma (Table 1).

**Table 1:** Participant occupation and relevant experience with the intervention.

ID	Occupation	Experience with the intervention
P1	Researcher clinician	Has delivered the intervention to women who have a history of sexual trauma
P2	Researcher clinician	Involved in the conceptual and clinical development of the intervention
P3	Clinical psychology student	Has delivered the intervention to refugees in Sweden and the United Kingdom
P4	Intern	Has observed the intervention being delivered and worked alongside those who have translated the intervention protocol
P5	Clinical psychology student	Is preparing to use the intervention in a laboratory setting in the near future
P6	Researcher	Has been part of clinical training meetings for the intervention and meetings concerning its development
P7	Researcher clinician	Has delivered the intervention to frontline hospital staff in the emergency department and intensive clinical care
P8	Researcher clinician	Has delivered the intervention to refugees in the United Kingdom
P9	Researcher clinician	Has delivered the intervention to parents of children who have been recently admitted or discharged from the pediatric intensive care unit
P10	Researcher clinician	Has helped create and design the intervention and has delivered it to emergency department patients
P11	Clinical psychology student	Is preparing to use the intervention in a laboratory setting in the near future
P12	Clinician	Has delivered the intervention to patients in the emergency department

#### Qualitative Content Analysis

The qualitative content analysis generated three main themes, each with a number of subthemes.

#### Theme 1: Potential Benefits of the Digital Materials for Remote Delivery

Although participants were not probed directly to describe potential benefits of the digital materials, all expressed positive comments about their utility in promoting remote delivery of the intervention.

##### (i) Animations Effectively Convey Content

Participants reported that intrusive memories were clearly explained and that the illustrations should help target users to understand the symptoms:

I really liked when, [it was showing] it can be like a camera picture, and it had the picture, and the camera sounds, [or] it can be like a movie, and it had a movie reel... I think that really clarified that difference... that was really effective.

Participant 8

Instructions for the imagery-competing task were said to be clearly conveyed, and the animations may help target users understand the instructions even more so than face-to-face delivery:

I think it’s really visually good that you have like, the little brain on the side, and ...you can show the blocks inside the brain to really like, show that you have to think about it, and it becomes very clear how mental rotation works because you have that little brain on the side.

Participant 5

One participant noted that the videos would be useful because target users can go back and rewatch them at any time to review the information. This participant works with parents of children in the pediatric intensive care unit, a target population that is often under acute stress and/or sleep deprivation and may benefit from the opportunity to revisit instructions:

In fact, you could probably be more helpful over video because parents can pause and stop and rewind.

Participant 9

##### (ii) Appropriate Style and Pace

General positive feedback was given on the style of the animation for depicting what can often be a difficult topic:

It’s simple, but respectful at the same time, if that makes sense, and without seeming like a cartoon, but doing that in a thoughtful but very accessible way. So, I think that balance has been struck beautifully.

Participant 2

The videos were also praised for their steady pace and frequent pauses to allow target users to process the content. Feedback suggested that the new materials hold initial promise regarding target user *engagement* because of the animation style and pacing. Participants described the digitalized materials as being something that, “you can pay attention to*...* which is good” (Participant 8) and said that they were presented at a pace that allows for time to process the materials (Participants 4 and 7). A few participants mentioned that it was perhaps too slow at times and that for certain populations such as frontline hospital staff, the pace of the video may need to be increased because of their busy work schedules.

##### (iii) Animations Convey Empathy

The depiction of intrusive memories was said to convey empathy for target users, perhaps even more than a face-to-face explanation typically would:

It explains the concept. But I think it also shows participants that you understand what “I'm” going through. And I think that's really, really powerful.

Participant 2

One participant commented on the facial expression of the character, stating that, from her experience with patients in the emergency department, the character showed an appropriate amount of distress to depict the experience of an intrusive memory, without being over the top:

...she looked sad or like she was experiencing stress when she got the intrusive memories. But she didn't look like, that scared.

Participant 12

##### (iv) Digital Materials Foster Standardization Across Studies

Participants reported that the materials could be used across studies for different groups of target users. For example, if used for research purposes, a standardized approach to intervention delivery would increase experimental control and allow more direct comparison of results across studies:

I think that videos would be great, not only for remote, but also for a more like, improved generalisation of the intervention in both an experimental and clinical setting.

Participant 6

##### (v) Quizzes Are Useful Checks for Understanding

It was not required for participants to answer the quiz questions, only to review them. All participants who did submit answers (9 of 12) scored 100%. Participants expressed that the quiz questions overall captured the important concepts from the video and should be useful to check target user comprehension:

[They are] good because it checks out the learning they’ve had from like, watching the video, making sure they understand what they’ve watched... They’ve covered the main points of what you’re aiming to get out of it.

Participant 8

#### Theme 2: Refining Materials Before Implementation

In addition to the aforementioned positive feedback, participants suggested some changes to help refine the digital materials before being implemented for pilot testing with target users.

##### (i) Modifications for Clarity of Symptom Explanation

Some participants suggested drawing a clearer distinction between intrusive memories and related concepts such as rumination, by including more specific examples (eg, stating that they are not the same as thinking, “I wish I could have done something differently”).

One participant said that the video did not clearly cover flashbacks, that is, a dissociative experience where the individual feels or acts as if the trauma event is happening again [2]:

I don't really think that the video captured flashbacks... if it's explaining intrusive memories, it doesn't have to be explaining flashbacks. But if I were to show this to a participant, I think I would pause and then want to add it can also be a flashback.

Participant 3

Two participants suggested that a particular aspect of the animation was unclear: the three droplets of blood that depict an example of an intrusive memory. They suggested that it be replaced by a red puddle instead of droplets; therefore, it is more apparent that the character has a memory involving blood:

Why [are] there some drops, red drops?... I [didn’t] realise what it was.

Participant 4

Some small modifications were also suggested to clarify the wording of two quiz questions, such as when multiple answer options could be conceived as technically correct, as opposed to only one, as intended.

##### (ii) Modifications to Clarity of Intervention Instructions

Some participants expressed that some aspects of Tetris gameplay were not addressed in the video, such as what to do when *Game Over* is reached and that target users tend to have questions about all the functions of the game, including that the game speeds up as time goes on:

...it would be helpful to inform participants that the Tetris will speed up. And so, I know that kind of was asked by participants a couple of times as they were going through the intervention. They were saying, you know, “it's getting faster!”

Participant 9

It was also suggested that the video should have more emphasis on *mental rotation*, as it is a key component of the intervention, and that this could be at the end of the video to promote a recency effect. One participant mentioned that the video should state that target users are playing Tetris with *special instructions* (instead of just playing Tetris) to reduce the chance that a target user who is already familiar with the game will dismiss the instructions as unnecessary.

#### Themes 3: Key Challenges for Remote Delivery

During the interviews, participants also raised some key challenges that should be considered in the shift toward full digitalization and remote delivery of the intervention.

##### (i) Overcoming the Lack of Real-Time Communication

Main challenges raised for remote delivery were how to allow target users to ask questions and how to give real-time feedback or encouragement. This was said to be especially important for target users who have severe dissociation symptoms (ie, the intrusive memory leading to the person losing touch with the here and now). One participant described that from her experience of working with refugees with complex trauma history, guidance when completing the intervention can help keep these users stay *grounded* during gameplay. Furthermore, users may lose focus on the *mental rotation* instructions after a while and need reminders to do this:

The one kind of drawback that I can think of is that, if the participant is wanting to ask a question, or wanting to pause and get that kind of straightaway feedback to answer questions, that might not be possible... The researcher might remind the participant to keep doing the mental rotation, especially when it starts moving quite quickly. I find that people then say, “Oh, I just totally lost track of what I was doing... I was just trying to focus on not filling up the screen.”

Participant 7

Some participants raised the question of how quizzes will be implemented, specifically how to proceed if target users answer any of the questions incorrectly. It was suggested that immediate feedback could be provided within the platform or an answer key provided.

##### (ii) Additional Videos to Advance Remote Delivery

Participants were asked in each interview if they had recommendations for additional videos that could help advance remote delivery. All participants had at least one suggestion for videos to explain other aspects of the procedure that are normally described face-to-face. This could include how to fill in the intrusive memory diary to assess the target symptom or how to instruct participants to create a list of their intrusive memories for the memory reminder part of the intervention. It was suggested that videos could help instruct how to access the game and troubleshoot common technical problems but that such instructions might differ across studies, for example, when playing Tetris across different platforms. Several participants raised the need to translate the materials for future planned studies that will include participants from other countries, such as women in Iceland, health care staff in Sweden, and refugees across Europe.

##### (iii) Maintaining a Sense of Bidirectional Relationships

Some participants raised the importance of fostering a sense of a bidirectional relationship between the intervention provider and target user, as this may help with retention:

I think, when we digitalise something we need, as we are losing, you know, the interpersonal component. ... we need to make sure we kind of fill that gap. Relationship still is the key for every treatment.

Participant 4

### Evaluation and Implementation of Feedback

A total of 9 suggested changes were considered to be *Must have* [29] and were modified accordingly; these are described in Table 2. Other feedback not prioritized as a *Must have* was not directly implemented at this time; however, in some cases, it may be addressed in future iterations and/or study and site-specific protocols (the complete table of feedback and assigned MoSCoW ratings is given in Multimedia Appendix 4). For instance, it was suggested that the word *trauma* should be avoided in the materials, as some target users may be nonclinical participants who receive the instructions in relation to watching stressful film materials, and the word *trauma* could be misleading in that setting. It was decided that this suggestion was too study/site specific to be implemented in the current materials but may be applied to other materials for those studies.

**Table 2:** Suggested changes to the digital intervention materials that were categorized by the research team as *Must have* and subsequently implemented.

Suggested change during interview	Changes made to the digital intervention materials
Add that intrusive memories come without warning or are *involuntary* somewhere in the video	Changed the summary sentence at the end of the video from “pop up in your mind without warning” to “pop suddenly into your mind when you don’t want them to” to emphasize that they are involuntary
Clarify the distinction between rumination and intrusive memories, by adding examples of the former, such as thinking “what could I have done differently?” or “why did this happen to me?”	Added to the script for the video on intrusive memories, “they are also NOT the same as thinking in words, like ‘Something awful happened to me’”
Clarify the three red drops of blood image, perhaps by drawing a red puddle instead, as it was difficult to distinguish what this was	Animation in the video was altered to represent blood more clearly
Emphasize mental rotation more as it is thought to be critical to the intervention’s efficacy	Changed the script and rerecorded to say, “for this intervention to work, the most important thing for you to do is focus on the blocks that are coming up next”
Annunciate the word *brain* more clearly and others a little clearer	Both videos have both been revoiced by a different person, with emphasis on clear annunciation
Say in the video that this is playing Tetris with special instructions	Change the name of the video when it is presented to target users to “Tetris with Special Instructions”
For the “How to Play Tetris” quiz, the first question, two of the possible answers are technically correct	Changed the first alternative for the first question from “Score as many points as possible” to “Getting as many orange blocks as possible” so that latter is more clearly incorrect
Some phrasing from the video is not included in the “How to Play Tetris” quiz, like saying “planning in your mind’s eye”	Changed the first option of the last question from “Visualise and rotate them in your head” to “Visualise and rotate them in your mind’s eye”
The wording of one question in the “What Are Intrusive Memories?” quiz, where the one option says, “intrusive memories usually take the form of*...*” “a headache” can be true for some participants and should be changed	Changed the third option of the second question stating that intrusive memories usually take the form of “a really high fever” to be more clearly incorrect

## Discussion

### Principal Findings

In line with our first aim, we obtained and analyzed qualitative feedback on digital intervention materials, which showed that participants were generally positive about the potential benefits of the digital materials for remotely delivering the intervention. The animated videos were thought to effectively explain the target symptom and to instruct participants in the imagery-competing task. Participants noted that the digital materials were engaging and could likely be used across different demographics of target users, highlighting their potential to standardize delivery of the intervention across studies. Relatively minor suggestions were made to clarify some aspects of the materials and, in line with our second aim, we systematically implemented these changes, making 9 modifications to the videos and quizzes. Participants also raised some key challenges in moving forward with successful remote delivery of the intervention, such as overcoming the lack of real-time communication between the researcher and target user. We discuss how these considerations will shape the next steps of intervention development.

### Comparison With Previous Work

Participants expressed several potential benefits of digital materials that align with previous findings. For example, participants reported that the videos effectively conveyed the content, and previous research has shown that animations with visual cues can be an effective medium for communicating scientific concepts, sometimes leading to better retention than static images [39]. Videos also allow content to be presented to the viewer in bite-size pieces, minimizing the chance of cognitive overload [40].

A number of specific suggestions for refining the materials were also raised, some to clarify the explanation of intrusive memories. Psychoeducation is a staple of most posttrauma interventions, and encouraging participants’ understanding of symptoms is a core aspect of psychoeducation [41]. This reflects the need for delivering crystal-clear explanations of the target symptom, as participants raised in our study. Suggestions were also made to clarify the instructions for the imagery-competing task. Previous work has shown that target users who have difficulties with the technological aspects of digital interventions are less likely to engage with them [42], and user errors may negatively affect the relationship between the target users and the health care provider [43], again highlighting the need for clear and straightforward digital materials.

Finally, participants raised key challenges to address in advancing the remote delivery of the intervention. These challenges generally align with those encountered in previous research on digital interventions. Participants raised the question of how to address target user questions if they arise during the session and the need to give real-time feedback. In digital interventions, for individuals with chronic physical health conditions, for example, remote interventions have been found to both empower and motivate participants; however, when feedback is given too often, patients may feel overly reliant on their health care providers [44]. Thus, a balance must be reached between the amount of guidance provided by the intervention providers and the level of independence offered to target users.

### Next Steps of Intervention Development

These findings will help guide the next steps of intervention development. One of the next steps, suggested by participants, will be to create additional study-specific videos. For instance, videos could help to guide participants through the intervention, from baseline assessment to accessing the Tetris game to keeping track of the target symptom. Such instructions will often differ across studies, depending on the target group and the platform used for intervention delivery. These study-specific videos may therefore be more practical to make as simple film clips of the researcher giving instructions, rather than highly polished animations that would only be used for a single study. Such clips of the researcher may also help users feel that the material is more personalized, as videos with real people could foster a more personal connection with the study. On the basis of participants’ feedback, the next steps should also include implementing real-time feedback to the quiz questions via the platform to provide encouragement or correct misunderstandings.

Critically, the next step is then to obtain feedback from different populations of target users, who do not have previous experience with the intervention, on the feasibility and acceptability of the digital materials. Given that many digital interventions are hampered by low rates of *engagement* and high *attrition* [26,27], finding ways to boost engagement and minimize dropout will be crucial. Both objective and subjective measures of engagement should be captured from target users in the next stages of pilot testing [27]. Depending on the functionality of the platform used for digital delivery, it may be possible to capture objective metrics such as frequency and duration of self-administered intervention usage. Subjective measures could include self-reported questions about aspects of engagement such as attention, interest, and affect [27] as well as open-ended questions about the intervention experience (eg, suggestions for what would make the intervention more acceptable or easier to complete). Pilot testing will help to inform the extent of real-time support, if any, needed from the researcher for successful delivery of the intervention to target users. By *real-time support*, we mean any guidance provided from the researcher remotely during a session (eg, via telephone or secure video link), such as to clarify task instructions, provide encouragement, or direct participants toward next steps. Real-time researcher support should thus be included as part of the protocol in the next stages of studies, before moving toward a more self-guided (and potentially more scalable) intervention. Findings will help to identify aspects of digital materials that need modification to minimize the amount of support and promote participant independence. For example, the technological literacy of different groups may affect the degree of technical support needed, such as troubleshooting videos, as suggested by some participants in this study.

Regarding quizzes, it will be important to examine (and then optimize) the difficulty level for target users. Target users in different groups should be able to understand the content and answer all quiz questions correctly. Ideally, this brief comprehension check should be in line with face-to-face procedures; quizzes should be a helpful *light-touch* repetition of the most crucial parts of the instructions. We intend to program the quizzes in such a way that if a user selects the wrong option, they will be informed of the correct answer in real time via a friendly pop-up message, similar to how a researcher would clarify any misunderstandings in person. Pilot testing will help to refine these procedures and determine whether such corrective feedback is sufficient or if, for example, target users need to rewatch the videos before proceeding to the intervention.

Many previous works have demonstrated the value of obtaining target user feedback to improve intervention materials, which is core to PBA [28]. For example, feedback from target users on a digital intervention for PTSD in women veterans highlighted the need to incorporate additional advice in the material, such as on seeking social support [45]. Piloting our digital materials on trauma survivors will likely raise issues to address in future iterations of the intervention that are not yet considered here. Finally, an intervention must not only be engaging and accessible to target users but must also be effective. After initial pilot testing with target users and further refinement of the digital intervention based on those findings, the next step should be rigorous testing of efficacy, starting, for instance, with single-case series designs [46], before scaling up to larger randomized controlled trials.

### Strengths and Limitations

This study has several strengths. First, systematic adjustments were made to the materials by first obtaining feedback from nontarget users of the intervention [29]. This should save time and resources by addressing any current flaws in the materials before direct piloting with trauma survivors. Second, interviews were carried out by KD, who had recently joined the research team and was not involved in the creation of the videos, perhaps allowing for more frank feedback on the materials from participants. Third, the sample included a range of professional backgrounds, from psychology students with cursory knowledge of the intervention, to experienced clinicians actively delivering the intervention, which should have led to a wider perspective on digital materials. However, we note an important limitation in that all participants were women from Europe; a more demographically diverse sample would have produced a broader perspective on, for instance, cultural sensitivities to keep in mind during intervention development.

### Conclusions

Digital interventions hold exciting promise for the dissemination of evidence-based treatments globally; however, they must be developed in a careful, stepwise manner to be engaging and effective for target users [27]. We have shown that clinicians, researchers, and clinical psychology students are confident in the use of digital materials to deliver a brief intervention to reduce the number of intrusive memories of trauma. Important challenges remain, such as overcoming the lack of real-time communication with target users. By refining the digitalized intervention materials, this study lays the groundwork for the next step of intervention development: pilot testing with trauma survivors.

## Appendix

Multimedia Appendix 1 Guiding principles for development of the digitalized intervention materials.

Multimedia Appendix 2 Interview guide.

Multimedia Appendix 3 Qualitative content analysis codes.

Multimedia Appendix 4 Suggested changes and MoSCoW categorization. MoSCoW: Must Have, Should Have, Could Have, Would Like.

## Abbreviations

MoSCoW: Must Have, Should Have, Could Have, Would Like
PBA: person-based approach
PPI: patient and public involvement
PTSD: posttraumatic stress disorder
