# Supplementary material for: Digitalizing a Brief Intervention to Reduce Intrusive Memories of Psychological Trauma: Qualitative Interview Study
Source: JMIR Ment Health. 2021 Feb 22;8(2):e23712. doi: 10.2196/23712 (PMC7939943; doi:10.2196/23712)
Supplement: Multimedia Appendix 1 [file mental_v8i2e23712_app1.pdf]

Supplementary Materials: Multimedia Appendix 1

Guiding Principles for Development of the Digitalised Intervention Materials

Guiding Principles for the “What Are Intrusive Memories?” Materials

**Video and Quiz Design Objectives – “What Are Intrusive Memories?”**	**Key Features**
Know how to accurately identify what an intrusive memory is and what it is not in a way that can be understood by the general public	• Identify the definition of an intrusive memory.<br>• Understand what an intrusive memory is not (e.g. rumination, “thinking in words” about the event).
Understand the phenomenology of intrusive memories	• Understand they can be vivid, short, broken-up, and fleeting.<br>• Understand that they most commonly take the form of visual memories, but may include other sensory sensations as well (e.g. sounds, smells).<br>• Understand how they may be triggered (e.g. by seeing something similar on the news, or in real life that reminds one of the event).<br>• Understand the functional impact of intrusive memories (e.g. that they can impact social functioning such as making it harder to keep in touch with friends, and concentration).
To provide a low-cost, scalable explanation and visual demonstration of what intrusive memories are in a thoughtful and generalisable way	• Limit the need for researcher/clinical explanation of what intrusive memories are.<br>• Visually showing intrusive memories can be related to distress, by adding a character with a concerned facial expression.<br>• Creating a character that is gender ambiguous.<br>• Choosing broad yet not disturbing visual examples of trauma memories (e.g. a picture of a car to represent a car accident, or three droplets of blood to represent a violent memory).

Guiding Principles for the "How to Play Tetris" Materials

**Video and Quiz Design Objectives – "How to Play Tetris"**	**Key Features**
Be able to understand the basic concept of the game	• Place blocks in a way that will clear the rows.<br>• How to drag and move the blocks.<br>• How to rotate the blocks.
Know how to use mental rotation	• Plan where to place the blocks by really twisting and turning them in the mind's eye to decide where they should go next.<br>• Refer to the 3 upcoming blocks displayed on the right side of the game.
Understand that importance of playing Tetris with these special instructions	• Process over points – using mental rotation and planning is more important than scoring points.<br>• It is important to play the game carefully, to allow time for planning.
To provide a low-cost, scalable explanation and demonstration of how to play the game in adherence to previous study protocols	• Limit the need for researcher/clinician explanation of how to play the game and what is meant by mental rotation.<br>• A brief yet informative overview of the special game instructions that includes examples.<br>• Reminders of the main goal of game.
