# Supplementary material for: Digitalizing a Brief Intervention to Reduce Intrusive Memories of Psychological Trauma: Qualitative Interview Study
Source: JMIR Ment Health. 2021 Feb 22;8(2):e23712. doi: 10.2196/23712 (PMC7939943; doi:10.2196/23712)
Supplement: Multimedia Appendix 2 [file mental_v8i2e23712_app2.pdf]

Supplementary Materials: Multimedia Appendix 2

Interview Guide

Feedback on videos – Researchers/Clinicians/Students

Language:____

Participant category:

Researcher/Clinician_______ Student ________

Experience delivering the intervention:________________________

Interviewer: ____

Date:____

ACTIONS		Done
Explain the background. Adapt information according to participant category	Moving towards remote delivery of a psychological intervention that has been delivered face to face in a stepwise manner.<br><br>The first step is to create short videos explaining pieces of intervention.<br><br>Videos will be gradually integrated into the intervention, which will still be delivered by a researcher.<br><br>The final goal is full remote delivery.	
May I use your feedback? For example, may I quote you in my thesis or other papers. Your name will not be used.		
May I record this session? Zoom records both the audio and video aspects of the call, but only the audio will be saved and used for the purpose of writing up my thesis. I would start recording after you have watched the video.<br>Is it okay if I record this session?		

Send the dropbox link and let the participant look at the video		
Ask the following questions		

Name of video: "How to Play Tetris"

**Questions**	Feedback
Tell me about your first impression of the video.	
Please describe your thoughts about using this instead of a face to face explanation.<br><br>Tell me how you think a participant would perceive this.	
Please describe you thoughts on the video quiz questions.<br><br>How do you think a participant might perceive them?	
Please describe if you found any aspects of this video helpful or unhelpful in any way.<br><br>And if so, how?	
Please describe if you found anything to be missing or redundant in this video.<br><br>**Probes**<br>*Can you tell me more about that?*<br><br>*In what way is that?*	
Please describe any thoughts or ideas you may have around potential ways to improve this video.<br><br>**Probes**<br>*Can you tell me more about that?*<br><br>*In what way is that?*	

How do you mean?	
Please describe anything we should consider in regards to your specific study population?	
Please let us know if you have any suggestions for what we can do next, such as what other video instructions might be helpful,<br><br>or if you any concerns about our next steps in digitalising the intervention and other parts of the research that you were think work or not work in this digital video-format, and why.	
Now I would like to show you four short quiz questions we have created for those who will use the intervention, that relate to the content of the video.<br><br>Send link/ask them to view/answer questions.<br>https://www.surveymonkey.com/r/MYLW5C9	
Please tell me your thoughts about the quiz questions.	
Please describe if there anyway you think that these questions could be improved.	
Now we will move on to the next video. Please watch the video, if you haven't already.<br><br>Let me know when you are ready and they can move on to the interview.	

Name of video: “What are Intrusive Memories?”

**Questions**	Feedback
Tell me about your first impression of the video.	
Please describe your thoughts about using this instead of a face to face explanation.<br><br>Tell me how you think a participant would perceive this.	
Please describe you thoughts on the video quiz questions.<br><br>How do you think a participant might perceive them?	
Please describe if you found any aspects of this video helpful or unhelpful in any way.<br><br>And if so, how?	
Please describe if you found anything to be missing or redundant in this video.<br><br>**Probes**<br>*Can you tell me more about that?*<br><br>*In what way is that?*	
Please describe any thoughts or ideas you may have around potential ways to improve this video.<br><br>**Probes**<br>*Can you tell me more about that?*<br><br>*In what way is that?*<br><br>*How do you mean?*	

Please describe anything we should consider in regards to your specific study population?	
Please let us know if you have any suggestions for what we can do next, such as what other video instructions might be helpful,<br><br>or if you any concerns about our next steps in digitalising the intervention and other parts of the research that you were think work or not work in this digital video-format, and why.	
Now I would like to show you four short quiz questions we have created for those who will use the intervention, that relate to the content of the video.<br><br>Send link/ask them to view/answer questions: https://www.surveymonkey.com/r/FMZCTMR	
Please tell me your thoughts about the quiz questions.	
Please describe if there anyway you think that these questions could be improved.	
Please let me know if you have any additional comments or concerns you would like for us to know:	

Notes/observations about the interview:
