# Supplementary material for: Digitalizing a Brief Intervention to Reduce Intrusive Memories of Psychological Trauma: Qualitative Interview Study
Source: JMIR Ment Health. 2021 Feb 22;8(2):e23712. doi: 10.2196/23712 (PMC7939943; doi:10.2196/23712)
Supplement: Multimedia Appendix 3 [file mental_v8i2e23712_app3.pdf]

Supplementary Materials: Multimedia Appendix 5

Codes derived from qualitative content analysis of participant feedback

Feedback on the "What are intrusive memories?" video and quiz

Positive	Suggested changes
**General**<br>• A benefit with using videos<br>• Good combination with voice and animation<br>• Soft, gentle voice<br>• Friendly<br>• Calm<br>• General positive feedback<br>• Captures interest/attention<br>• Less concerns now with experience<br><br>**Standardisation**<br>• Applicable across populations<br>• Offers a more standardised protocol<br>• Good explanation for researcher/clinician too<br><br>**Animation**<br>• The visuals are very helpful<br>• Liked that it was a person of colour<br>• Liked that the person is gender neutral-more relatable<br>• Liked the motivational mouse<br>• Shows empathy<br>• Respectful<br>• Thoughtful<br>• Liked the camera example<br>• Liked the phone example<br>• Good, simple trauma examples<br>• Liked the character<br>• Good facial expression of character<br>• Love the graphics<br>• Liked the thought bubbles<br>• Liked the movie example<br>• Liked the day/night example<br>• Liked the car crash example<br><br>**Explanation/Clarity**<br>• Good explanation of sensory aspects<br>• Good example of functional impact	**Animation**<br>• Clarify blood example<br>• Mouse could be bigger<br>• Images and words should appear together<br>• Emphasise vividness of memory examples<br>• Emphasise fleetingness of memory examples<br>• Work visual not clear<br>• Maybe show more distress<br>• Add bullet points at the end<br><br>**Explanation of Intrusive Memories**<br>• Clarify between rumination and thinking over<br>• Clarify more what it is not<br>• Add that they come without warning<br>• Clarify 'dissociation'<br>• Clarify difference between re-experiencing<br>• Use simpler language<br>• Add something to normalise intrusive memories<br>• Generalise the concentration example<br>• Add "without triggers/triggers you are not aware of"<br>• Add that they can be short and fragmented<br>• Add more specific concentration examples<br>• Doesn't cover flashbacks<br>• Rumination visual not clear<br>• Add something about distress<br>• Add something about physical symptoms<br>• Explain dissociation more<br><br>**Quizzes**<br>• Wording of quiz<br>• Mention Tetris with Special Instruction<br><br>**Other**<br>• More tailored trauma event<br>• Add a general list of trauma examples<br>• Too slow pace at time

• Good example of disconnect<br>• Very clear/concise<br>• Easier to understand<br>• Very clear/helpful voice<br>• Good explanation of intrusive memories<br>• Good explanation of what may happen<br>• Good recap at the end<br>• Good differentiation between thinking and rumination<br>• Good balance of information<br><br>**Length and Speed**<br>• Pace allows for time to process materials<br>• Good overall pace<br>• Brief questions<br><br>**Quizzes**<br>• Checks for understandings<br>• Good quiz questions<br>• Good to have quiz questions	• Mouse took away focus<br>• Don’t use the word trauma<br>• Say "negative experience" instead

Feedback on the “How to play Tetris with mental rotation” video and quiz

Positive	Changes
**General**<br>• A benefit with using videos<br>• Captures attention<br>• General Positive Feedback<br>• Good combination with voice and animation<br>• Calming<br>• Educational<br>• Less distraction<br>• Keeps your attention<br>• Soft, gentle voice<br>• Human Element<br>• Fun (Tetris quiz)<br><br>**Standardisation**<br>• Offers a more standardised protocol<br>• Applicable across populations<br><br>**Animation**<br>• Colourful<br>• Design of the blocks<br>• Good animation with the brain<br>• Good, middle-ground example<br>• The visuals are very helpful<br><br>**Explanation/Clarity**<br>• Clear division of instructions<br>• Cleary explains the instructions<br>• Very clear/concise<br>• Very clear/helpful voice<br>• Easier to explain what the game is<br>• Easier to understand<br>• Good explanation of the game<br>• Good focus on next 3 pieces<br>• Good, useful recaps<br>• Good, clear focus on mental rotation<br>• Good wording / simple language<br><br>**Length and Speed**<br>• A good length of time/short<br>• Good overall pace<br>• Time saving<br>• Pace allows for time to process the materials	**Explanation of the Tetris game**<br>• Start with basic instructions first<br>• Add instruction to turn off notifications<br>• Address breaks and game play duration<br>• Address that points/score does not matter<br>• Address that there is a "hold" function<br>• Address the speed of the game, there is no need to rush<br>• Address what to do after "game over"<br>• Mention something about practicing<br>• More repetition needed<br>• Order of the example is wrong<br>• Show what the three blocks on the side mean earlier<br><br>**Explanation of Mental Rotation**<br>• Add another example<br>• Emphasise mental rotation more<br>• End with Mental Rotation explanation<br>• Enforce mental rotation over points<br><br>**Animation**<br>• Personify the brain more<br>• Adjust colour contrast with blocks on brain<br><br>**Other**<br>• Mention "Tetris with special instructions"<br>• Address other things in protocol<br>• Clarify audience for quiz questions<br>• Cut down on long gaps<br>• Hard to hear the word "brain"<br>• Language barrier/translations needed<br>• Too slow of a pace at times<br>• Dialect may be a bit difficult<br><br>**Quizzes**<br>• Wording of quiz<br>• Add "planning in mind's eye" in Tetris quiz

Feedback more generally on next steps for advancing digital remote delivery

Suggestions for Next Video/Steps	General Concerns
**Technological Needs**<br>• Explain setup<br>• Explain/troubleshoot general problems<br>• Explain/troubleshoot tech problems<br>• Need to address using different devices/platforms<br><br>**Other parts of the intervention**<br>• Explain the difference between flashbacks and intrusive memories<br>• How to create a list for intrusive memories<br>• Need to have video on filling in diary<br>• Reminder of memory recall<br><br>**Other**<br>• Prepare a handout with instructions<br>• Make videos downloadable<br>• Provide answer key to quiz questions<br>• Add character to all videos<br>• Translation<br>• Voice over for questions	**Communication with participant**<br>• If participants don't understand<br>• Ensuring a bidirectional relationship<br>• Checking for understanding<br>• How participants will ask questions<br>• Chance to practice online with clinician<br>• Can't check if paying attention<br>• Can't visually see if participants understand<br>• Face-to-face might be better for trauma survivors<br>• Making sure instructions are followed<br>• Need context for participants<br>• Start with having researcher there<br>• The need for giving feedback to participants<br>• The need to give participant encouragement<br><br>**Other**<br>• Following the order of the videos<br>• Need to think a lot about explanation of intrusive memory video<br>• No access to Wi-Fi for some<br>• Subtitles are not enough<br>• The style might not be taken seriously<br>• Overall logistics<br>• May not get the quiz right<br>• Should reach out to participants<br>• Make sure participants are listening<br>• Participants finding the time and place<br>• Participants who can't read<br>• What platform will they view these on
