# Supplementary material for: Digitalizing a Brief Intervention to Reduce Intrusive Memories of Psychological Trauma: Qualitative Interview Study
Source: JMIR Ment Health. 2021 Feb 22;8(2):e23712. doi: 10.2196/23712 (PMC7939943; doi:10.2196/23712)
Supplement: Multimedia Appendix 4 [file mental_v8i2e23712_app4.pdf]

Supplementary Materials: Multimedia Appendix 6

Suggested changes to the materials and their MoSCoW categorisation

Suggested change	Reason for change (based on PBA)	Priority (MoSCoW)	Agreed change (if no change agreed, explain why)
"How to play Tetris with mental rotation" video			
- Add another example of mental rotation.	**Consistent with Common Guiding Principles**: to support *autonomy* and *competence* by providing another example.<br><br>**Useful across populations**	**Should have** – this normally has to be re-explained and demonstrated several times in face-to-face delivery, since this is a new and specific way of playing Tetris, and also a vital part of the intervention.	No. Instead, have an option or instruction to repeat (watch again) in the future.
- Reverse the order of this example, so that it is the green block that is addressed first and then the orange one.	—	**Would like** – a more logical example could make it easier for participants to understand how to plan ahead better.<br><br>- This counter intuitive example enhances the MR. Showing that it takes a while, look at *upcoming block* first, even if there is another one falling at the same time. Better to focus on the upcoming best than the current one.	No. The current order is suffice in that starting the MR explanation shows viewers that *this* is the focus of the game during the intervention, rather than focusing on the basic instructions first.
- Add eyes to the brain, or draw a head or body around the brain.	—	—	No. Not necessary.
- Adjust the colour contrast of the blocks on the brain.	—	—	No, too specific/not necessary.
- Emphasis mental rotation more.	**Important for behaviour change** – modification is likely to impact behaviour change or a precursor to behaviour change.<br><br>**Consistent with Common Guiding Principles.** (autonomy, competence)<br><br>**Repeated by several participants.**<br><br>**Useful across population**	**Must have** – this is considered to be a crucial part of the intervention for it be effective.	Yes.

- Change the order of the video and start with the basic instructions first.<br>e.g. show what the three blocks on the side mean earlier in the video.<br><br>-The mental rotation explanation and example should be at the very end of the video.	**Repeated by several participants.**<br><br>**Useful across population**	**Would like** –<br>(Basic instructions first, people may dismiss the whole video)	No. The "mental rotation" reminder is stated at the end already.
-Add an instruction on a reminder to turn off your mobile phone notifications, and to have less distractions around you.	**Important for behaviour change** – modification is likely to impact behaviour change or a precursor to behaviour change.<br><br>**Consistent with Common Guiding Principles.** (autonomy, competence)<br><br>**Repeated by several participants.**<br><br>**Useful across populations**	**Should have** – these need to be addressed as taking breaks for example or using the hold function may alter the effectiveness of the game play.<br><br>-Adding something to script like "you need to play in this particular way, for this long, fully focused, make sure you're not interrupted"	Addressed in procedure, so will be addressed but not in video.
- Mentioned what to do if you take a break during the game, and how long you should play for.	**Important for behaviour change** – modification is likely to impact behaviour change or a precursor to behaviour change.<br><br>**Consistent with Common Guiding Principles.** (autonomy, competence)<br><br>**Repeated by several participants.**<br><br>**Useful across populations**	**Should have** – these need to be addressed as taking breaks for example or using the hold function may alter the effectiveness of the game play.<br><br>-Adding something to script like "you need to play in this particular way, for this long, fully focused, make sure youre not interupted"	Addressed in procedure, so will be addressed but not in video.
- Add an instruction on the "hold" function.	**Important for behaviour change** – modification is likely to impact behaviour change or a precursor to behaviour change.<br><br>**Consistent with Common Guiding Principles.** (autonomy, competence)	**Would like** – but not crucial for this intervention.	No. This is not something that is brought up by a lot of participants and may be quite intuitive (i.e. participants can catch on to this function and do not need explicit explanation for this.)

	**Repeated by several participants.**<br><br>**Useful across populations**		
- Add that the game does speed up over time, but one should not rush.	**Important for behaviour change** – modification is likely to impact behaviour change or a precursor to behaviour change.<br><br>**Consistent with Common Guiding Principles.** (autonomy, competence)<br><br>**Repeated by several participants.**<br><br>**Useful across populations**	**Would like** – but not crucial for this intervention.	No, this is not exactly necessary for this intervention.
- Address that points do not matter.<br><br>- Say in the video that this is playing Tetris *with special instructions*.	**Important for behaviour change**<br><br>**Consistent with Guiding Principles**<br><br>**Consistent with Common Guiding Principles**<br><br>**Uncontroversial and easy**<br><br>**Repeated by Several Participants**<br><br>**Useful across populations**	**Should have,** this is an important part of game play for this intervention, to not focus on points. Mentioning this could lead to users focusing more on using mental rotation during game play instead.<br><br>“not important to score, but play in this particular way” + addresses below comment too	No, not in video but should be addressed in written protocol of the studies, or in hand-outs.
- Cut down on some of the long pauses in the video.	—	—	No. Perhaps this may be true for hospital staff, but for most of the target groups, the pace is appropriate. The video is also still very brief as it is.
- Annunciate this word more clearly and others a little clearer.	**Consistent with Guiding Principles** – explaining how to use mental rotation and plan ahead<br><br>**Consistent with Common Guiding Principles** – to promote *competence*<br><br>**Repeated by several participants.**<br><br>**Useful across population**	**Must have** at least for the word “brain” – this could make it clearer for some viewers but not necessary, since there is an picture of a brain in the video to help support this.	Yes.

- Audio and subtitles in different languages should be made and applied to the video.	**Important for behaviour change**<br><br>**Repeated by several participants**<br><br>**Useful across populations**	**Must have** – depending on the target user, this must be done in order for the intervention to be effective in changing a participant's behaviour (given what we know about the evidence base).	Yes.
"How to play Tetris with mental rotation" quiz			
- Change the first option with something else as this is also technically correct, even though this is not the main goal of this intervention.	**Consistent with Guiding Principles** (being able to identify the main objective of the game)<br><br>**Consistent with Common Guiding Principles** (to promote competence)<br><br>**Repeated by several participants**<br><br>**Uncontroversial and easy**<br><br>**Useful across populations**	**Must have** – this should be changed so that users will not be confused and the correct answer, which touches on using mental rotation and planning, is further emphasises as the correct answer.	Yes.
- Add "planning in the mind's eye" to one of the options, to the relevant quiz question.	**Important for behaviour change**<br><br>**Uncontroversial and easy**<br><br>**Useful across populations**	**Must have** – adding this phrase may help users engage more in the quiz, as they may remember this phrasing from the video and then select the correct answer based on this.	Yes.
- Remove the mouse figure.	—	—	No. It would be more work to remove it. It helps to add a motivational and personal aspect to the videos, something that does need to be compensated for in digital interventions
"What are Intrusive Memories" video			
- Clarify this example more, perhaps by drawing a red puddle instead.	**Repeated by several participants.**<br><br>**Uncontroversial and easy (still animation)**	**Must have** – it could add to the clarity of the visualisation but would not be likely to impact behaviour change or understanding of the content of the video.	Yes.

		-maybe drops and small puddle, clearer. Should not be too “simple” but also not traumatising.	
- Add that intrusive memories come without warning, involuntary, or use some simpler language like this.	**Important for behaviour change** to address this.<br><br>**Uncontroversial and easy**	**Must have** – need to know what intrusive memories are, and word *involuntary* nicely and briefly describes them	Yes.
- Clarify dissociation and reexperiencing more. This may not be relevant for intrusive memories, where it says in the video, “it can feel you’re back in the situation”, these are not a part of intrusive memories?	—	—	No, as they are not the target symptoms for this intervention, but these symptoms plan on being mentioned in other materials, such as general psychoeducation on psychological trauma.
- Add something that normalises intrusive memories, such as “they are really common to experience after a traumatic event…”	**Consistent with Common Guiding Principles** – *provide a sense of relatedness*.<br><br>**Useful across populations**	**Could have** – would be useful, but not critical for behavioural change.<br><br>Convey somewhere else perhaps. Normalization more for psychoeducation	Not at this time, but in supplementary materials that are group/study specific.
- Add that intrusive memories can appear even without triggers you are aware of.	—	—	No. Too complex for this video.
- Add that intrusive memories can be short and fragmented	**Consistent with Guiding Principles** (to be able to identify what an intrusion is…)<br><br>**Consistent with Common Guiding Principles** – *competence*, that this is something the counts as an intrusion and should be recorder in the diary.<br><br>**Useful across populations**	**Would like** – Animation shows a piece of memory	No.<br>Another video will be made for diary, will keep that in mind though.
- Emphasise vividness of memory example by changing their colour gradient or make the memory appear in different levels of transparency.	**Repeated by several participants.**	**Would like** – it could add to the clarity of the visualisation but would not be likely to impact behaviour change or understanding of the content of the video – as it is already state they can be vivid.	No, it is already mentioned.

		(without changing script, just animation)	
- Add more on how examples distressing intrusive memories can be, and the physical symptoms one can feel, such as a stomach ache, when experiencing intrusive memories.	**Consistent with Common Guiding Principles** – sense of *relatedness*	**Would like** – not needed to support behaviour change, but could be useful if time and resources allow.	No, physical symptoms often go along with distress.
- Include more tailored trauma examples by animation, and/or naming some common examples based on previous study populations.	**Consistent with Common Guiding Principles** – sense of *relatedness*<br><br>**Repeated by several participants**	**Should have** – would be useful, but may not impact behavioural change.<br><br>In other study materials, should address more population specific things but not in the video/script.	No, not enough time to address this change. But a good consideration for the future, and/or perhaps in other supplementary materials, (e.g. as part of the recruitment flyer or other information materials.)
- Emphasise fleetingness of memory example, by making them appear very quickly and then disappear.<br><br>- Sync the audio with the animations more.	—	—	No. It is enough with what is in the script perhaps (review this though, as this comes up in later on again…)
- Change the work example to something more generalisable and relatable, such as working on a computer.	**Consistent with Common Guiding Principles** – sense of *relatedness*	**Would like** – not needed to support behaviour change, but could be useful if time and resources allow	No, because this work example is quite universal actually. Whether one is studying, or even just doing chores one may have to write things down.
- Add more on dissociation. For example, that some people may dissociate when playing Tetris during the intervention, or at other times.	**Important for behaviour change**<br><br>**Consistent with Guiding Principles**<br><br>**Consistent with Common Principles**	**Should have** – important, at least for those who have reported to experience this while playing Tetris. May be able to address this in a flyer/website/during check ins instead of including in the video.	No, other materials, very population/user specific.
- Add more specific examples for how intrusive memories can disrupt one’s concentration. For example, that they can when one is studying.	**Consistent with Common Guiding Principles** – sense of *relatedness*	**Would like –** would make it more useful perhaps and relatable for student participants, but not necessary.	No. The current examples are broad enough. Students would get additional information as part of lab study. And the video already addresses concentration.

- Clarify more between what is rumination and “thinking it over”, and intrusive memories. For example, also add that they does not include evaluative thoughts such as, “why did this happen to me?” nor thinking, “what could I have done differently?”, nor going home and describing the memory to another person.	**Consistent with Guiding Principles** – to identify intrusions<br><br>**Repeated by several participants**<br><br>**Useful across populations**	**Must have** – this is important for users to be able to determine what should be recorded as an intrusion and what should not be recorded in their diaries.	Yes.
- Show more distress in the character’s face, for other target groups.	**Consistent with Common Guiding Principles** (sense of relatedness)	**Would like** – not needed to support behaviour change.	No. Not needed – Guiding principle positive experience, don’t want it show too much distress.
- Add bullet points of the key phrases at the end of the video.	**Important for behaviour change**<br><br>**Consistent with Guiding Principles** (being able to identify intrusions)<br><br>**Uncontroversial (but maybe not easy**)<br><br>**Useful across populations**	**Should have -** having bullet points in the recap at the end could make it easier for viewers to understand the main take-aways of the video. It would be nice to see these bullet points along with the animation, but if this is not possible than it can be included in other supplementary information, such as a flyer.	No, this can be supplemented in other ways (i.e., as a hand-out) rather than editing the video.
- Speed up the pace at times, and/or remove some of the long pauses in between phrases.	**Repeated by several participants**	**Would like** – it could be useful, but the video is already brief (only 3 minutes long) and this is population specific.	No, keep the current pace. The video is already quite brief. (*May* be adjusted for groups like hospital staff in the future)/
- Take away the mouse.	—	—	No. Mouse will probably stay. See previous comment regarding the mouse.
- Use the word “negative experience” instead.	**Important for behaviour change** (including precursors, acceptability, engagement)	**Should have** – for non-clinical viewers, this would be a more appropriate change because they may not identify as a “trauma” survivor, or may be primed by using the word trauma, which may affect their behaviour during the intervention phase. This could also be implemented in another way, such as stating to these participants beforehand that the video uses the word “trauma” which does not entirely apply to them.	Won’t change video but, as this is group specific.<br>Instead, this can be addressed in the lab script for the same video.

"What are Intrusive Memories" quiz			
- Change this option, because some users have reported that they get headaches when they have intrusions.	**Consistent with Common Guiding Principles** (competence, autonomy)<br><br>**Uncontroversial and easy**<br><br>**Useful across populations**	**Must have** – changing this option on the quiz would lead to less confusion for users who *do* have headaches with their intrusions.	Yes.
- Add audio to assist in answering the questions (e.g. a voice that reads the options, and assigns a colour to each option, so that the user can pick the answer without having to read text.)	**Consistent with Common Guiding Principles** (competence, autonomy)	**Would like** – too group specific.	No, as this is very specific to the individual
